# NUF2 Promotes Breast Cancer Development as a New Tumor Stem Cell Indicator

**DOI:** 10.3390/ijms24044226

**Published:** 2023-02-20

**Authors:** Yang Deng, Jiapeng Li, Yingjie Zhang, Hao Hu, Fujian Wan, Hang Min, Hao Zhou, Lixing Gu, Xinghua Liao, Jingjiao Zhou, Jun Zhou

**Affiliations:** Institute of Biology and Medicine, College of Life and Health Sciences, Wuhan University of Science and Technology, Wuhan 430081, China

**Keywords:** breast cancer, NUF2, cancer stemness

## Abstract

Multiple new subtypes of breast cancer (BRCA) are identified in women each year, rendering BRCA the most common and rapidly expanding form of cancer in females globally. NUF2 has been identified as a prognostic factor in various human cancers, regulating cell apoptosis and proliferation. However, its role in BRCA prognosis has not been clarified. This study explored the role of NUF2 in breast cancer development and prognosis using informatic analysis combined with in vivo intracellular studies. Through the online website TIMER, we evaluated the transcription profile of NUF2 across a variety of different cancer types and found that NUF2 mRNA was highly expressed in BRCA patients. Its transcription level was found to be related to the subtype, pathological stage, and prognosis of BRCA. The R program analysis showed a correlation of NUF2 with cell proliferation and tumor stemness in the BRCA patient samples. Subsequently, the association between the NUF2 expression level and immune cell infiltration was analyzed using the XIANTAO and TIMER tools. The results revealed that NUF2 expression was correlated with the responses of multiple immune cells. Furthermore, we observed the effect of NUF2 expression on tumor stemness in BRCA cell lines in vivo. The experimental results illuminated that the overexpression of NUF2 statistically upregulated the proliferation and tumor stemness ability of the BRCA cell lines MCF-7 and Hs-578T. Meanwhile, the knockdown of NUF2 inhibited the abilities of both cell lines, a finding which was verified by analyzing the subcutaneous tumorigenic ability in nude mice. In summary, this study suggests that NUF2 may play a key role in the development and progression of BRCA by affecting tumor stemness. As a stemness indicator, it has the potential to be one of the markers for the diagnosis of BRCA.

## 1. Introduction

Breast cancer is the most prevalent cancer among women worldwide, with the fastest expanding rate among all types of female cancers. In 2020, the number of new patients reached 2.26 million [1]. Breast tumors are generally divided into three main subtypes based on hormone status: estrogen receptor (ER), human epidermal growth factor receptor 2, and HER2 (HER2) [2,3], together with some rare subtypes, including luminal subtypes that express ER and/or progesterone receptor (PR), HER2-positive subtypes that overexpress HER2, and triple-negative breast cancer (TNBC) [2,4]. Triple-negative breast cancer accounts for 10–20% of the multiple subtypes [5]. For decades, chemoradiotherapy and endocrine therapy have been effective, but the recurrence and metastasis of breast cancer remains a major clinical challenge [6]. By analyzing the differentially expressed genes in tumor cells, doctors are now trying to target specific markers or proteins to kill the cells or diagnose the disease early.

NUF2 is a structural protein in a highly conserved protein complex connected to the centromere. It is a component of the NDC80 kinetochore complex. The human NUF2 gene is structurally comparable to the yeast NUF2 gene. During meiosis, the expression of the NUF2 gene in yeast is ceased when the centromere loses contact with the spindle. This suggests that it could be responsible for the way in which chromosomes divide [7,8,9,10]. In the latest study, through the analysis of the TCGA database and the GEO database, it was found that NUF2 was upregulated in prostate cancer (PC) [11], non-small-cell lung cancer (NSCLC) [12], hepatocellular carcinoma (HCC) [13,14,15], esophageal squamous cell carcinoma (ESCC) [16], and kidney renal clear cell carcinoma (KIRC) [17] and was partially verified in patient tissue samples or cell lines. In small-cell esophageal carcinoma (SCLC), Liu et al. found that NUF2 is involved in tumor-stem-cell-related pathways [18]. Furthermore, there is increasing evidence to suggest that NUF2 is involved in the immune infiltration of a variety of tumors [11,19,20,21]. These results demonstrate that NUF2 plays a crucial role in tumor cells, while the mechanism and role of NUF2 in BRCA progression remain unspecified.

The present study aimed to investigate the expression of the NUF2 gene in BRCA and its influence on tumor stemness. Through gene enrichment and PPI network analysis, we found that NUF2-related pathways, as well as the associated proteins, were enriched and linked mainly to cell proliferation, and through correlation analysis, we ventured to speculate that NUF2 may affect tumor stem cell competence. Finally, through in vitro experiments, we found that NUF2 significantly promoted the stem-cell-like phenotype of BRCA cell lines. Through in vivo experiments, NUF2 was found to promote the ability of BRCA cells to become tumorigenic in the subcutis of mice. Theoretically, these findings could aid in the early identification of BRCA patients and the development of innovative, individualized therapeutics.

## 2. Results

### 2.1. Expression Pattern of NUF2 from a Pan-Cancer Perspective

**Figure 1:**
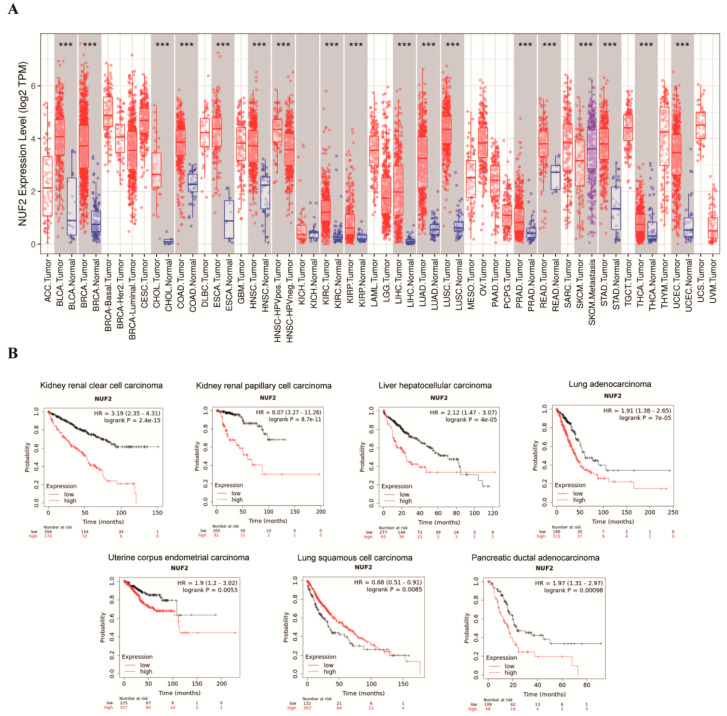
Expression Pattern of NUF2 from a Pan-Cancer Perspective. (**A**) Pan-cancer analysis of NUF2 expression based on the TIMER database. (**B**) Survival curves of KIRC, KIRP, LIHC, LUAD, LUSC, and PAAD, excepting breast cancer, with divergent NUF2 expressions. *** *p* < 0.001.

NUF2 mRNA expression profiles of pan-cancer patients were downloaded from the online tool TIMER. Data from the Kaplan–Meier Plotter website were retrieved for a patient prognosis evaluation. The data analysis showed that NUF2 mRNA was highly expressed in 16 types of human cancer (Supplementary Table S1) (Figure 1A), among which 7 types of cancer with abnormal NUF2 mRNA expression profiles presented with a significantly differential prognosis, namely KIRC (kidney renal clear cell carcinoma), KIRP (kidney renal papillary cell carcinoma), LIHC (liver hepatocellular carcinoma), LUAD (lung adenocarcinoma), UCEC (uterine corpus endometrial carcinoma), LUSC (lung squamous cell carcinoma), and PAAD (pancreatic ductal adenocarcinoma) (Figure 1B). Except for LUSC, patients in the high-NUF2-expression group had a worse prognosis.

### 2.2. Elevated NUF2 Expression and Worse Prognosis in Patients with Breast Cancer

**Figure 2:**
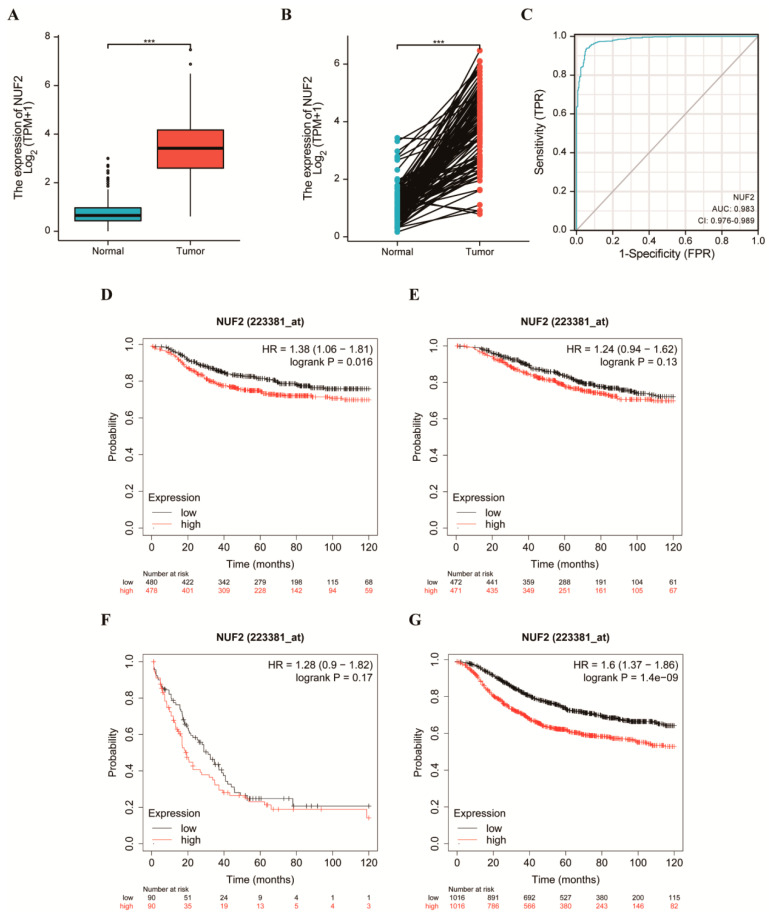
Elevated NUF2 expression and worse prognosis in patients with breast cancer. (**A**) The mRNA expression level of NUF2 in 1109 breast cancer patient and normal tissues. (**B**) The mRNA expression level of NUF2 in 112 BRCA patient samples and their matched adjacent normal samples. (**C**) The ROC curve of NUF2 in BRCA. (**D**–**G**) The Kaplan–Meier survival curve showed that the DFMS(D), OS(E), PPS(F), and RFS(G) of breast cancer patients with high NUF2 mRNA expression were shorter than those of breast cancer patients with low NUF2 mRNA expression. *** *p*  <  0.001.

In total, 1109 cases of breast cancer and 113 adjacent normal tissue data were downloaded from TCGA. The expression profile was constructed to detect the expression of NUF2 in breast cancer. High NUF2 expression was found in the majority of the BRCA tissue samples (Figure 2A). Among them, 224 cases were paired, and the expression of NUF2 was increased at a statistically significant level (Figure 2B). The ROC curve of NUF2 was analyzed to investigate the diagnostic significance of NUF2 in differentiating breast cancer samples from normal samples (Figure 2C). The AUC value of NUF2 was 0.983, and the 95% confidence interval (95% CI) was 0.976–0.989. These results suggest that NUF2 may be a biomarker that can distinguish tumors from normal tissues. The online Kaplan–Meier Plotter tool was used to plot the distant metastasis-free survival (DMFS), overall survival (OS), post-progression survival (PPS), and relapse-free survival (RFS) curves of NUF2 (Figure 2D–G). The results showed that higher expressions of NUF2 had a worse prognosis in terms of the DMFS and FRS, and although there was no significant difference in the *p*-value between the OS and PPS, it was consistent with the previous findings in terms of the overall trend. Differences in the DMFS [HR = 1.38 (1.06–1.81), logrank *p* = 0.016] and RFS [(HR = 1.6 (1.37–1.86), and logrank *p* = 1.4 × 10−9] curves were significant. The results were not significantly different in terms of the OS [HR = 1.24 (0.94–1.62), logrank *p* = 0.13] and PPS [HR = 1.28 (0.9–1.82), or logrank *p* = 0.17] curves. The overall trend indicated that high NUF2 expression correlated with a worse prognosis.

### 2.3. Clinicopathological Features of Breast Cancer with Respect to the NUF2 Expression Level

**Figure 3:**
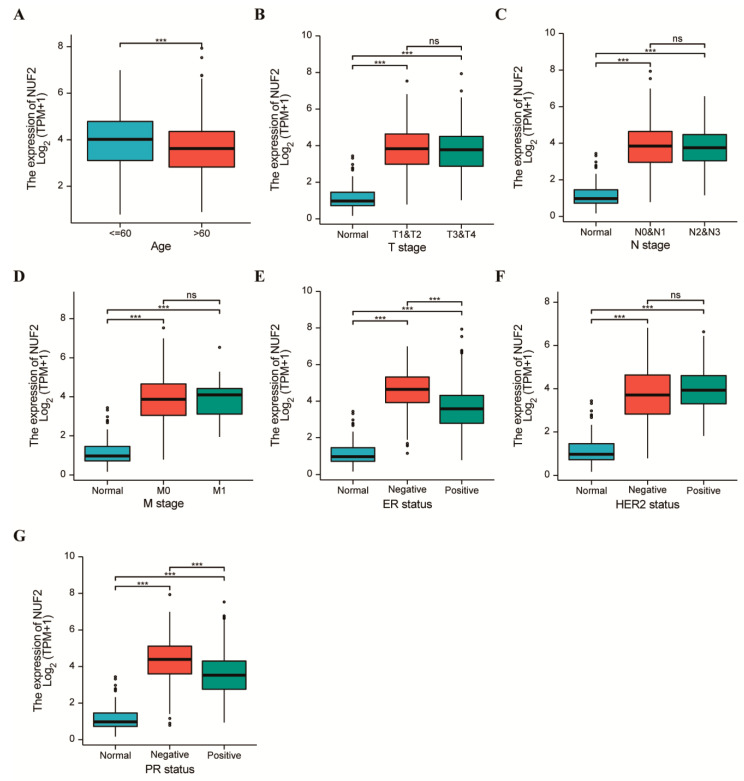
Clinicopathological Features of Breast Cancer with respect to the NUF2 Expression Level. (**A**) The relationship between the NUF2 mRNA expression level and age. (**B**–**D**) There was a significant correlation between NUF2 mRNA expression and the T stage, N stage, M stage of BRCA. (**E**–**G**) In the three subtypes of breast cancer, the NUF2 mRNA expression level was related to the PR, ER subtype, and HER subtype, but the PR, ER-negative NUF2 expression was higher. *** *p*  <  0.001. ns showed no significant difference.

We used the XIANTAO online tool to map the correlation between NUF2 expression and the pathological stage of BRCA patients. Additionally, the Mann–Whitney U test and logistic regression analysis were used for the analysis, which aimed to evaluate the relationship between NUF2 mRNA expression and the clinicopathological features of the breast cancer samples. The results shown in Figure 3A demonstrated that NUF2 expression was significantly lower in the samples of patients aged >60 years than in those aged ≤60 years. As shown in Figure 3B–D, the expression of NUF2 was correlated with the T stage, N stage, and M stage (*p* < 0.001). However, there were no significant differences between each stage. In the three BRCA subtypes (ER, PR, HER2), NUF2 expression was found to be higher in ER, PR-negative breast cancer. The difference was significant (*p* < 0.001) (Figure 3D–G). These results suggest that NUF2 was involved in the hormone receptor levels. Since the efficacy and prognosis of endocrine therapy for breast cancer were closely related to the hormone receptor expression level, NUF2 may be a biomarker for the diagnosis of BRCA.

### 2.4. Identification and Functional Enrichment of NUF2 Co-Expressed Genes

**Figure 4:**
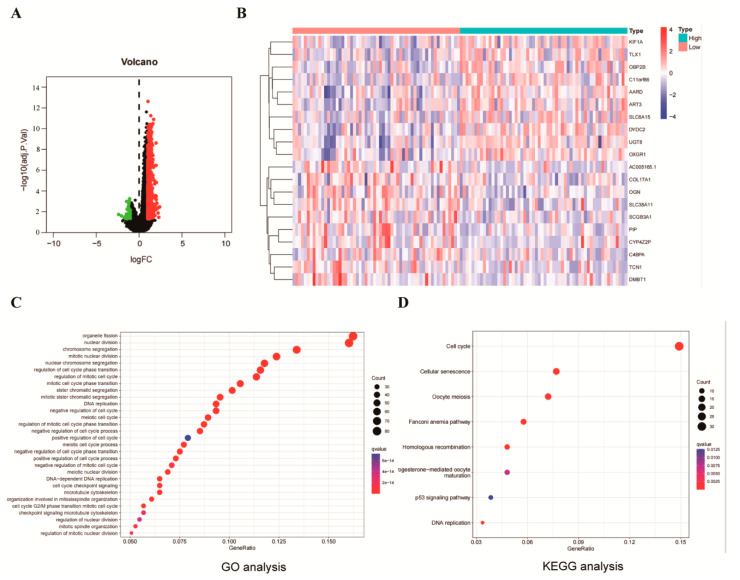
Identification and Functional Enrichment of NUF2 Co-Expressed Genes. All the data were collected from the TCGA-TNBC database. (**A**) The volcano plot of the co-expressed genes. (**B**) Heat map of the top 10 NUF2 co-expressed genes. (**C**) The top 30 co-expressed genes in the GO functional enrichment analysis of NUF2. (**D**) The top 8 KEGG pathways in the enrichment analysis of the NUF2 co-expressed genes.

Gene set enrichment analysis was used to investigate the potential mechanism of NUF2 in regulating breast cancer. All the collected TCGA-BRCA data were first normalized and transformed using log2 (X + 1). Using the R program with the limma package, the co-expressed genes were screened. The findings indicated the identification of 712 co-expressed genes. In total, 676 genes were elevated (logFC > 2), and 36 genes were downregulated (logFC < 2) (Figure 4A). Figure 4B depicts the top 10 genes exhibiting co-expressed gene associations. The GO enrichment analysis of the co-expressed genes indicated that organelle fission, nuclear division, chromosomal segregation, mitotic nuclear division, and nuclear chromosome segregation were the top five bioactive enrichment sites (Figure 4C). Similarly, the top five pathways identified in the enrichment analysis of the co-expressed gene KEGG pathways on the co-expressed genes were the cell cycle, cellular senescence, monocyte meiosis, Fanconi anemia pathway, and homologous recombination (Figure 4D). Overall, we found that the main pathway in which NUF2 is involved in breast cancer is cell proliferation, and NUF2 promotes cell proliferation.

### 2.5. Correlation between NUF2 and Tumor Stemness Markers

**Figure 5:**
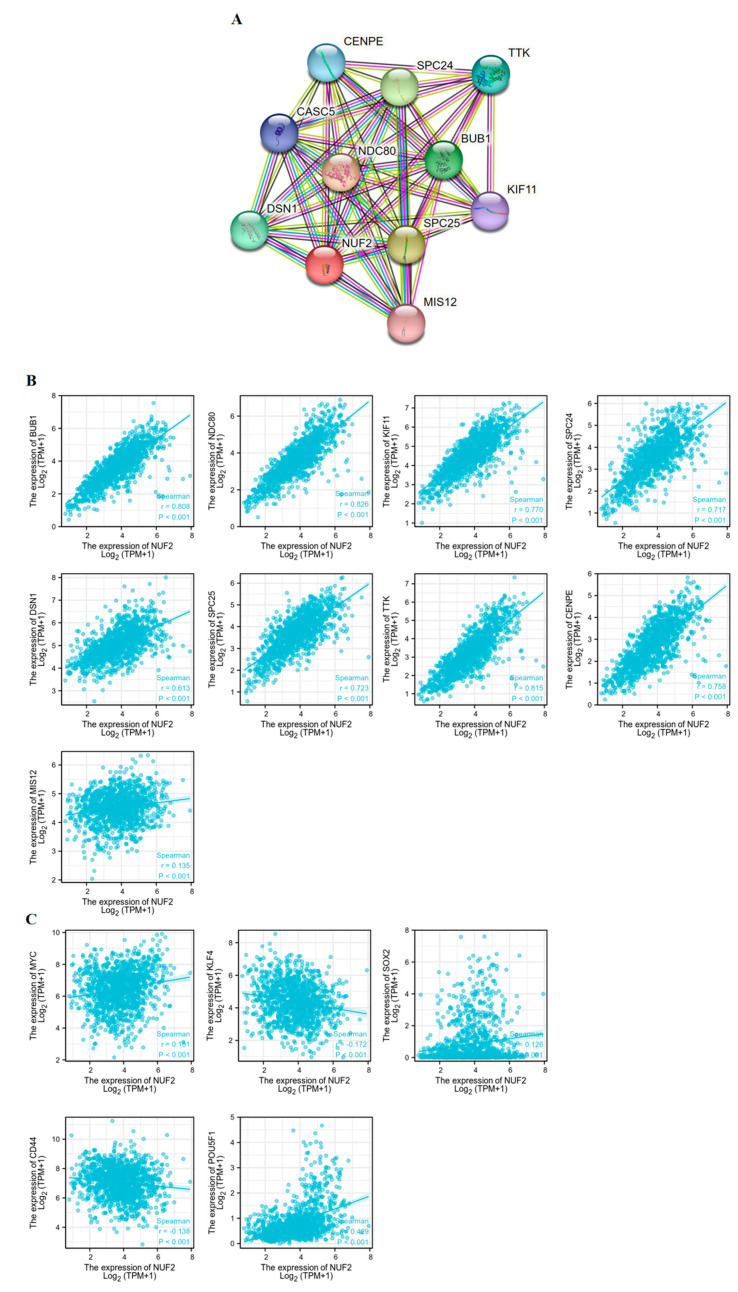
Correlation between NUF2 and Tumor Stemness Markers. (**A**) The top 10 genes correlated with NUF2 in the protein–protein interaction network. (**B**) Correlation between the mRNA expression level of NUF2 and related genes. (**C**) Correlation between the NUF2 mRNA expression level and tumor stem cell markers.

The STRING online tool was used to construct a PPI network and map the NUF2-related genes. The top 10 genes which showed the strongest correlations with NUF2 are presented in Figure 5A. We then used the XIANTAO online website to obtain association scatter plots of these nine genes with respect to NUF2 by analyzing the data of 1222 TCGA-BRCA cases, including BUB (r = 0.808, *p* < 0.001), NDC80 (r = 0.826, *p* < 0.001), KIF11 (r = 0.826, *p* < 0.001), SPC24 (r = 0.717, *p* < 0.001), DSN1 (r = 0.613, *p* < 0.001), SPC25 (r = 0.723, *p* < 0.001), CENPE (r = 0.758, *p* < 0.001), and MIS12 (r = 0.135, *p* < 0.001) (Figure 5 B). In the previous KEGG and GO analyses, it was found that NUF2 is involved in cell activities in the pathways related to cell division and proliferation. Most of the 10 most relevant genes were found to be related to cell proliferation. Therefore, we examined the correlation between NUF2 and tumor stemness markers (Figure 5C), and the results showed that NUF2 correlates with the following tumor stemness markers: MYC (r = 0.181, *p* < 0.001), KLF4 (r = 0.172, *p* < 0.001), SOX2 (r = 0.126, *p* < 0.001), CD44 (r = 0.138, *p* < 0.001), and POU5F1 (OCT4) (r = 0.429, *p* < 0.001). The results, thus, showed that NUF2 was positively correlated with most of the tumor stemness markers. Therefore, we can speculate that NUF2 may promote the development of tumor stemness.

### 2.6. The Effect of NUF2 on Immune Cell Infiltration in Breast Cancer

**Figure 6:**
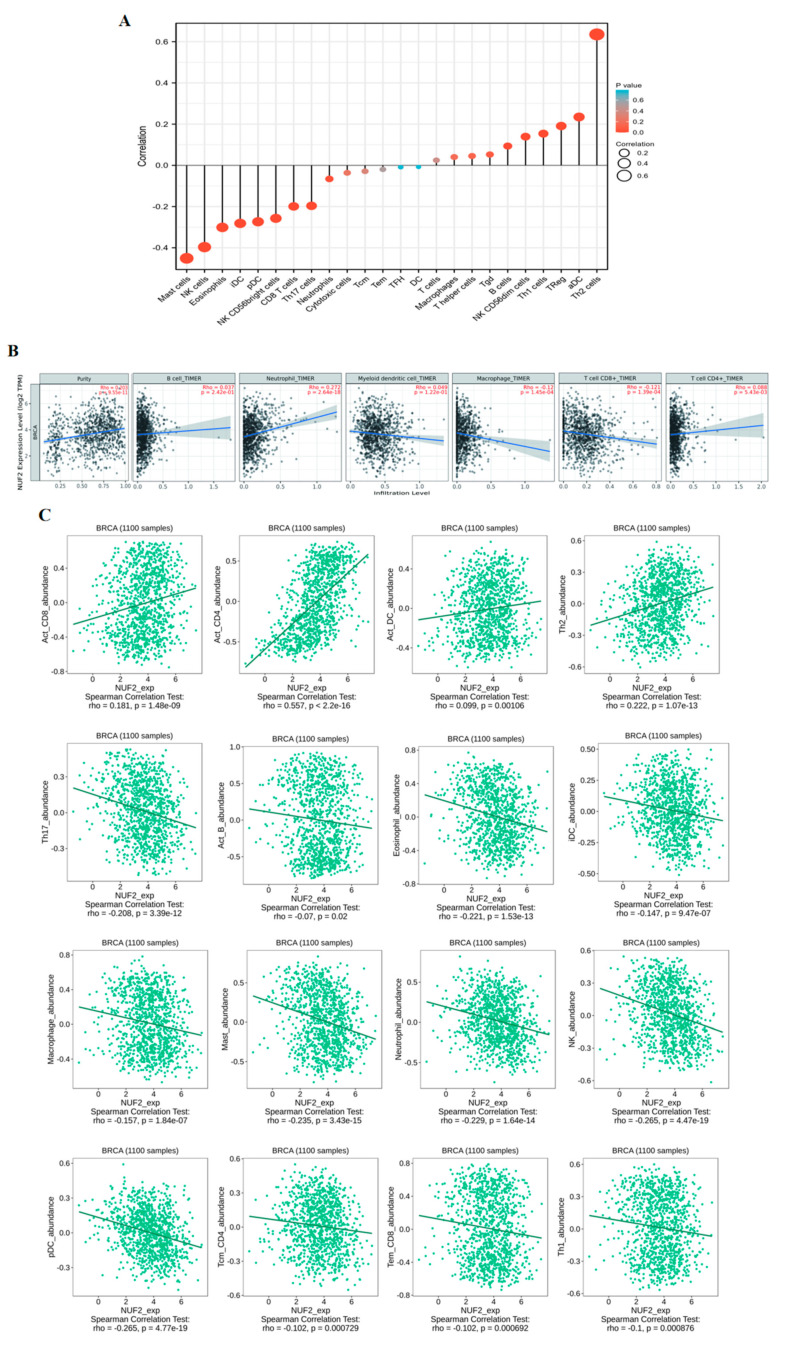
The Effect of NUF2 on Immune Cell Infiltration in Breast Cancer. (**A**) Correlation between NUF2 and 24 types of immune cells. (**B**) Scatter diagram of the correlation between NUF2 and 6 types of immune cells based on the TIMER online tool. (**C**) Using the XIANTAO tool, we mapped the correlations between NUF2 and 16 types of immune cells.

We then sought to investigate the relationship between NUF2 expression and immune infiltration. A total of 1222 TCGA-BRCA samples were selected to analyze the correlation between NUF2 and 24 immune cells through the online website XIANTAO (Figure 6A). The results showed that NUF2 was positively correlated with the Th2 cells, aDC, Treg, Th1 cells, and NK CD56dim cells. However, it was negatively correlated with the Mast cells, NK cells, Eosinophils, iDC, pDC, and NK CD56bright cells. This suggests that NUF2 may play a role in the immune response to BRCA. NUF2 expression was correlated with the Purity (r = 0.203, *p* = 9.55 × 10−11), Neutrophil (r = 0.272, *p* = 2.64 × 10−18), and Macrophage (r = −0.12, *p* = 1.45 × 10−4) on the online website TIMER (Figure 6B). Finally, scatter plots of NUF2 expression and 16 immune cells were plotted using the XIANTAO website. These cells were Act_CD8 (r = 0.181, *p* = 1.48 × 10−9), Act_CD4 (r = 0.557, *p* = 2.2 × 10−16), Act_DC (r = 0.099, *p* = 0.00106), Th2 (r = 0.222, *p* = 1.07 × 10−13), Th17 (r = −0.208, *p* = 3.39 × 10−12), Act_B (r = 0.07, *p* = 0.02), Eosinophil (r = −0.221, *p* = 1.53 × 10−13), iDC (r = −0.147, *p* = 9.47 × 10−7), Macrophage (r = −0.157, *p* = 1.84 × 10−7), Mast (r = −0.235, *p* = 3.43 × 10−15), Neutrophil (r = −0.229, *p* = 1.64 × 10−14), NK (r = −0.265, *p* = 4.47 × 10−19), pDC (r = −0.265, *p* = 4.77 × 10−19), Tcm_CD4 (r = −0.102, *p* = 0.000729), Tem_CD8 (r = −0.102, *p* = 0.000692), and Th1 (r = −0.1, *p* = 0.000876) (Figure 6C). These results suggest that NUF2 may promote the immune infiltration of breast cancer.

### 2.7. NUF2 Expression in Breast Cancer Cell Lines

**Figure 7:**
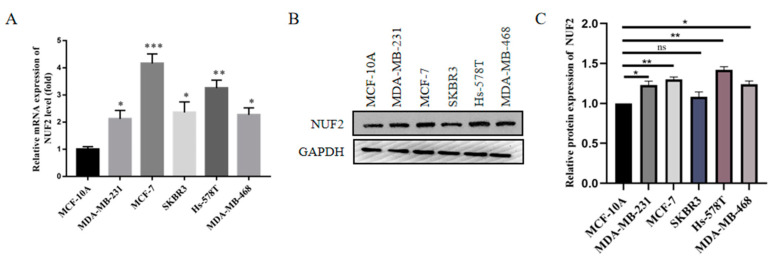
NUF2 Expression in Breast Cancer Cell Lines. (**A**,**B**) The expression of NUF2 in breast cancer cell lines detected by qRT-PCR and Western blot. (**C**) The Western blot results were quantified and normalized to GAPDH (*n* = 3). The data are presented as the mean  ±  SD, * *p*  <  0.05, ** *p*  <  0.01, *** *p*  <  0.001. ns showed no significant difference.

Through the qRT-PCR detection of breast cancer cell lines, we found that the expression of NUF2 was upregulated in breast cancer cell lines compared to normal breast epithelial cells, MCF-10A (Figure 7A). The same results were obtained by Western blot, and the expression level of the NUF2 protein was significantly upregulated in the breast cancer cell lines (Figure 7B,C).

### 2.8. The Efficiency of NUF2 Knockdown in MCF-7 Cells

After the stable knockdown of the NUF2 cell line, constructed in the MCF-7 cell line by lentiviral vector, the efficiency of NUF2 knockdown (sh-NUF2) was detected by qRT-PCR (Figure S1A). Additionally, the inhibitory effect of NUF2 sh-RNA on the protein level was verified by Western blot (Figure S1B).

### 2.9. Downregulated/Inhibited Tumor Stemness as a Result of NUF2 Knockdown in Breast Cancer Cell Lines

**Figure 8:**
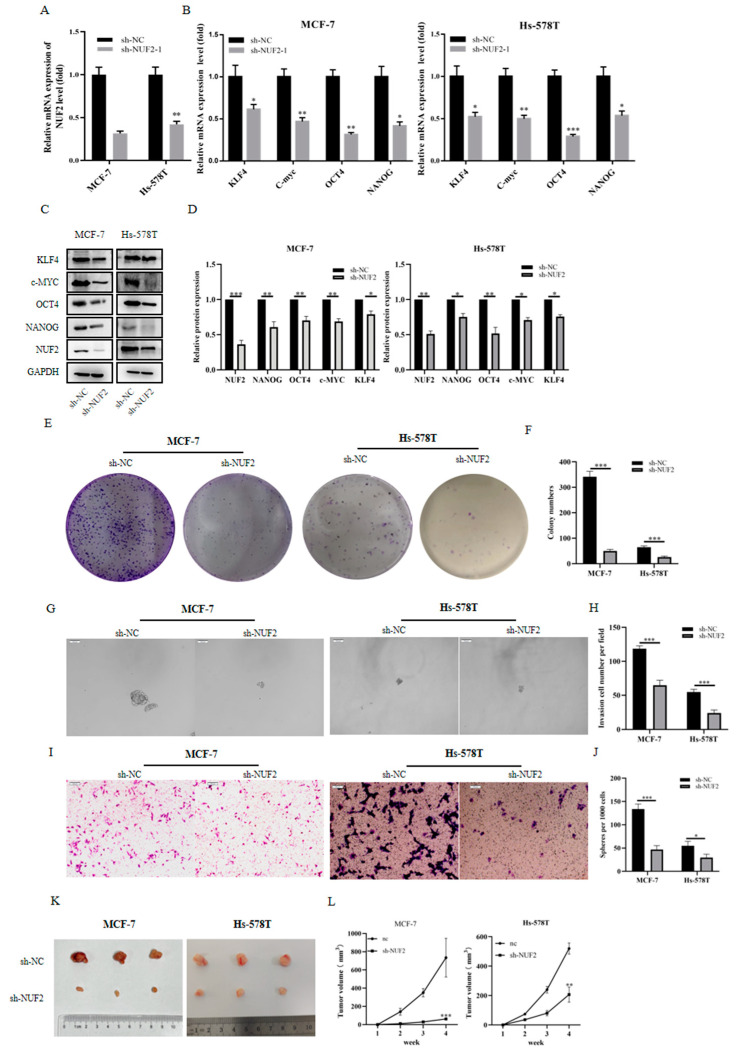
Tumor Stemness Downregulated by NUF2 Knockdown in Breast Cancer Cell Lines. (**A**) The mRNA expression level of NUF2 after knockdown. (**B**,**C**) The expression profiles of stem cell markers were detected by qRT-PCR and Western blot in NUF2 stable knockdown cell lines of MCF-7 and Hs-578T. (**D**) The Western blot results were quantified and normalized to GAPDH (*n* = 3). (**E**,**F**) The proliferative capacity of the MCF-7 and Hs-578 T cell lines after NUF2 knockdown was examined by clone formation experiments. The statistical analysis of the number of cell clones was performed in Image J (*n* = 3). (**G**,**H**) The ability to form tumor stem cells after NUF2 knockdown was examined by spheroid experiments. Spheroids in the entire dish were counted (scale bars, 100 μm). (**I**,**J**) The effect of the knockdown of NUF2 on the invasive ability. The statistical analysis of the number of cell clones was performed in Image J (*n* = 3). (**K**) Subcutaneous xenografts in nude mice. (**L**) Statistical analysis of the volume of subcutaneous xenografts (*n* = 3). The data are presented as the mean  ±  SD, * *p*  <  0.05, ** *p*  <  0.01, *** *p*  <  0.001.

We found that NUF2 expression was higher in the breast cancer cell lines MCF-7 and SKBR3 and the triple-negative breast cancer cell lines MDA-MB-231, Hs-578T, and MDA-MB-452 compared to the normal breast epithelial cells MCF-10A. In this work, the MCF-7 and Hs-578T cell lines were used in the knockdown assays. The effect of NUF2 mRNA expression knockdown was assessed by qRT-PCR (Figure 8A). qRT-PCR and Western blot were used to determine whether the knockdown of NUF2 reduces the expression of the tumor stemness markers (Figure 8B–D). Later, clone creation tests were conducted on the cell lines with the stable knockdown of NUF2. We discovered that the reduction in NUF2 expression significantly decreased the cell lines’ proliferative potential (Figure 8E–F). The spheroid experiment also demonstrated that NUF2 knockdown suppresses the proliferation of breast cancer tumor stem cells (Figure 8G–H). Finally, transwell experiments revealed that NUF2 knockdown inhibits the invasiveness of sh-NUF2 cell lines (Figure 8I–J). In vivo experiments were conducted on nude mice, in which two cell lines of sh-NC and sh-NUF2 were injected subcutaneously. The tumors were extracted 4 weeks later. The MCF-7 cells and Hs-578T cells with NUF2 knocked down in the nude mice were far less likely to turn into tumors (Figure 8K). Additionally, the tumors’ rate of growth was much slower (Figure 8L).

### 2.10. Tumor Stemness induced by NUF2 Overexpression in Breast Cancer Cell Lines

**Figure 9:**
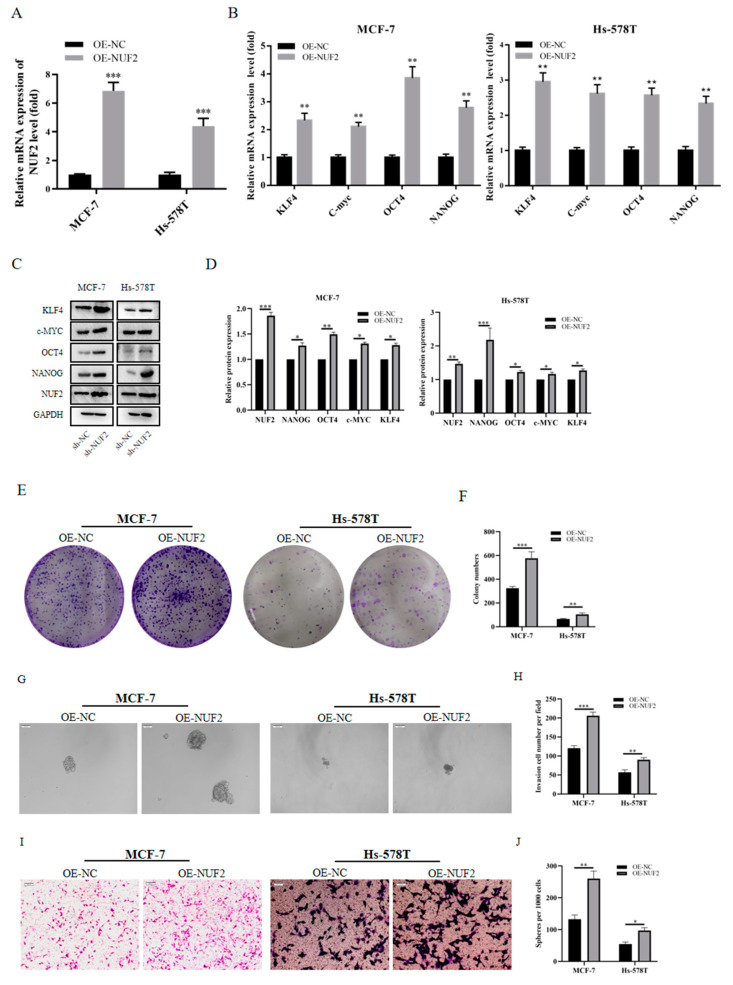
Tumor Stemness induced by NUF2 Overexpression in Breast Cancer Cell Lines. (**A**) The mRNA expression level of NUF2 after overexpression. (**B**,**C**) The expression profiles of the stem cell markers were detected by qRT-PCR and Western blot in NUF2 stable overexpression cell lines of MCF-7 and Hs-578T. (**D**) The Western blot results were quantified and normalized to GAPDH (*n* = 3). (**E**,**F**) Colony formation assays were used to assess the effect of NUF2 overexpression on the proliferative capacity of MCF-7 and Hs-578T cells. The statistical analysis of the number of cell clones was performed in Image J (*n* = 3). (**G**,**H**) The ability to form tumor stem cells after NUF2 overexpression. Spheroids in the entire dish were counted (scale bars, 100 μm). (**I**,**J**) Transwell assay detects the effect of NUF2 overexpression on the invasive ability of the MCF-7 and Hs-578T cell lines. The statistical analysis of the number of cell clones was performed in Image J (*n* = 3). The data are presented as the mean  ±  SD, * *p*  <  0.05, ** *p*  <  0.01, *** *p*  <  0.001.

After verifying that NUF2 knockdown inhibited the tumorigenicity of MCF-7 and Hs-578T, we sought to test whether the overexpression of NUF2 could similarly promote the capacity for tumorigenicity. Subsequently, NUF2 plasmids were constructed, and cell lines with a stable overexpression of NUF2 (OE-NUF2) were constructed. QRT-PCR and Western blotting analysis were used to assess the NUF2 overexpression efficiency (Figure 9A,B). Upon the Western blot examination of NUF2 overexpression, the expression of the given tumor stemness marker was also stimulated (Figure 9C,D). The clonogenesis experiments demonstrated that NUF2 overexpression promotes the cell proliferation capacity (Figure 9E,F). The spheroid experiment also showed that NUF2 overexpression promotes the proliferative capacity of tumor stem cells (Figure 9G,H). The transwell assay results demonstrated that OE-NUF2 could promote the invasive ability of breast cancer cells (Figure 9I,J).

## 3. Discussion

Breast cancer (BRCA) is the most common disease among women across the world. It is more common in European and American cultures than in Asian or African ones [22,23]. BRCA mortality is also the most common type of cancer-related death worldwide, accounting for 15% of all cancer deaths globally [24,25]. In response to the development of BRCA, many important signaling pathways have been identified to maintain tumor stemness, such as NOTCH, Wnt/β-catenin, STAT3, hedgehog, and other signaling pathways [26].

In this study, we found that NUF2 was highly expressed in 16 cancers and had prognostic significance in 7. The high expression of NUF2 in BRCA was found to be related to a worse prognosis. Our results are consistent with the findings reported in previous studies [27]. Through functional enrichment and PPI protein network analysis, we found that NUF2 may promote breast cancer tumor development by participating in cell division and cell cycle regulation. Therefore, based on a correlation analysis, we predicted that NUF2 might be involved in tumor stemness development and, subsequently, experimentally confirmed that NUF2 can, indeed, promote the expression of tumor stem cell indicators. This view is in line with that of Liu and Yu [13,18]. Similarly, although it is not confirmed that NUF2 can promote tumor stemness development, a large number of findings suggest that NUF2 can promote cell proliferation. Through KEGG analysis, we found that the influence of NUF2 on tumor stemness and invasion may be through the P53 pathway. These experimental results suggest that NUF2 may serve as one of the indicators of tumor stemness not only in breast cancer but also in other cancers. Finally, we also found that in BRCA, NUF2 may be involved in the immune infiltration of the tumor. Interestingly, NUF2 expression was positively correlated with Th1 and Th2 cells but negatively correlated with DC cells and NK cells, suggesting that NUF2 may play two roles in tumor immune infiltration, a finding that was first reported in BRCA but has since been reported in other cancers [11,19,20,21].

## 4. Materials and Methods

### 4.1. Research Workflow

In this study, we localized NUF2 expression to breast cancer in multiple cancer types and subsequently analyzed the expression, clinical diagnostic significance, and prognostic level of NUF2 in breast cancer. Then, the pathological characterization led us to develop the thesis that altered NUF2 expression occurs at the beginning of the cancer’s development. This argument was then further validated by GSEA and PPI network analysis. At the same time, an association between NUF2 expression and immune infiltration was also found. Finally, in vivo and in vitro experiments were performed to verify whether NUF2 is involved in tumor stemness.

### 4.2. TCGA Data Acquisition

We downloaded HTSeq-FPKM level 3 RNAseq data from the TCGA database (https://portal.gdc.cancer.gov/ (accessed on 7 June 2022)) [22], including 1109 breast cancer samples and 113 normal samples. Among these, 224 cases were successfully paired. The RNAseq data were converted from FPKM (fragments per kilobase per million) format to TPM (transcripts per million reads) format and log2-transformed.

### 4.3. NUF2 mRNA Expression Data and Prognosis

NUF2 expression in pan-cancer was mapped using the TIMER website. Then, we selected 1099 breast cancer samples and 292 normal breast samples from the XIANTAO platform (https://www.xiantao.love/ (accessed on 7 June 2022)) to analyze the NUF2 expression in BRCA. Among these cases, 112 breast cancer cases and 112 adjacent normal tissues were analyzed for the mRNA expression of NUF2. To examine the correlation of NUF2 with prognosis, we used the online Kaplan–Meier Plotter tool (https://kmplot.com/analysis/ (accessed on 8 June 2022)). “Auto select best truncation value” and “Basic type” were selected for the BRCA analysis, including the distant metastasis-free survival (DMFS) (958 cases), overall survival (OS) (943 cases), post-progression survival (PPS) (180 cases), and relapse-free survival (RFS) (2032 cases).

### 4.4. Pathway Analysis

The criterion for the differential gene screening was |log2 FC| > 2, and the adjusted *p* tool value < 0.05 was used as the standard. R was used with the clusterProfiler package (http://org.hs.eg.db (accessed on 8 June 2022)). The Gene Ontology (GO) and Kyoto Encyclopedia of Genes and Genomes (KEGG) pathways were examined using the enrichplot and ggplot2 programs.

### 4.5. Protein–Protein Interaction (PPI) Network Analysis

The protein–protein interaction networks were investigated using the STRING database (https://string-pb.org (accessed on 9 June 2022)) [28]. In this study, we used STRING to search for co-expressed genes and construct a PPI network [29]. Then, XIANTAO was used to collect the correlation coefficients between NUF2 and its co-expressed genes and tumor stemness markers.

### 4.6. Tumor Immune Assessment

TIMER (https://cistrome.shinyapps.io/timer/ (accessed on 9 June 2022)) [30] was used to establish the relationship between NUF2 and the immune response. Additionally, TIMER was used to determine the relationship between NUF2 expression and six types of immune infiltrates (B cells, CD4+ T cells, CD8+ T cells, neutrophils, macrophages, and dendritic cells) in breast cancer. The scatter plot of the correlation between NUF2 expression and immune cell infiltration was drawn. To increase the accuracy of the TIMER website, we also used the XIANTAO website for verification.

### 4.7. Cell Lines

This laboratory was the original owner of the breast cancer cell lines MCF-7 (subtype: Luminal A, ER+, PR+, HER2−) and SKBR3 (subtype: HER2, ER−, PR−, HER2+), whereas the China Cell Line Bank received the normal breast epithelial cell line MCF-10A. Wuhan Warner Biotechnology Co., Ltd. acquired the triple-negative breast cancer cell lines Hs-578T (subtype: Basal, ER−, PR−, HER2−), MDA-MB-468 (subtype: Basal, ER−, PR−, HER2−), and MDA-MB-231 (subtype: Basal, ER−, PR−, HER2−). MCF-10A was grown in MCF-10A-specific media (Procell, Wuhan, China). MDA-MB-231, SKBR3, Hs-578T, and MDA-MB-468 were grown in DMEM media (Gibco, Waltham, MA, USA) supplemented with 10% fetal bovine serum (Hyclone, Los Angeles, CA, USA). The cell lines were grown in an incubator containing 5% carbon dioxide with 37 °C saturated humidity.

### 4.8. qRT-PCR Analysis

The RNA simple Total RNA Extraction Kit (Qiagen, Darmstadt, Germany) was used to extract the total RNA from all the cells, and Nordoop2000 was used to quantify the concentration of the RNA, which was then corrected for further studies. Using a reverse transcription kit (Vazyme, Nanjing, China), the mRNA was converted to cDNA and employed as a template. The Applied BiosystemsTM SYBRTM Green technique was followed to set up the real-time fluorescence quantitative PCR reaction equipment (Thermo Fisher, Waltham, MA, USA). The PCR amplification reaction parameters were as follows: 40 cycles of 95 °C for 2 min, 95 °C for 10 s, and 60 °C for 30 s. Using the 2−ΔΔCt technique, the relative expression changes in NUF2 in each group were determined. The primers were as shown in Supplementary Table S2.

### 4.9. Cell Line Construction

The 10-cm plates were seeded with 5 × 106 cells/mL of logarithmic growth phase HEK-293T. Lipofectamine 2000 was then used to co-transfect the HEK-293T cells (Invitrogen, Waltham, MA, USA) with the previously created stable overexpression or knockdown plasmid of NUF2, the plasmid GAG (Invitrogen, USA), and the packaging plasmid VSVG (Invitrogen, USA). New DMEM cultures were added 24 h after transfection. The lentivirus solution was collected in a cryovial using a 0.45 μm filter after 72 h of transfection. Using a Lenti-Pac HIV RT-PCR Titration Kit (GeneCopoeia, Rockville, MD, USA), the viral load was determined. The virus was administered dropwise to the MCF-7 and Hs578T breast cancer cells cultured at 37 °C and 5% CO2 saturation in an incubator (Thermo Fisher, Waltham, MA, USA). After 72 h of transfection, the presence of puromycin in the cells was monitored continuously for 3 days. The chosen cells were inspected and submitted for examination. Supplementary Table S3 displays the sequence of sh-NUF2. Using an endotoxin-free plasmid extraction kit, the plasmid vectors were isolated (Cwbiotech, Beijing, China). The plasmid vectors were transfected using Lipofectamine 2000 (Invitrogen).

### 4.10. Western Blot

RIPA lysis buffer (Meilunbio, Wuhan, China) was used to lyse the cells and extract all of the proteins. The target protein concentration was measured using a BCA protein assay kit (Meilunbio, China) and standardized to 2 μg/μL, followed by the determination of the target protein expression using Western blot. Each sample was spiked with 30 μg of total protein. The BioRad ChemiDoc XRS+ imaging system was used to scan the ECL (Meilunbio, China) signal (BioRad, Hercules, CA, USA). The antibodies were KLF4 (Absin, Shanghai, China, 1:2000), OCT4 (Absin, Shanghai, China, 1:2000), NANOG (Absin, Shanghai, China, 1:2000), c-MYC (Absin, Shanghai, China, 1:2000), and GAPDH (Abclonal, Wuhan, China, 1:5000).

### 4.11. Clone Formation Assay

Trypsin-digested cells were collected in 15 mL sterile centrifuge tubes. A total of 500 cells were selected and placed on a six-well plate, followed by 2 mL of 10% FBS medium, which was thoroughly mixed. A new medium was introduced once every three days. The MCF-7 cells were grown in culture for 7 days, whereas the Hs-578T cells were grown for 14 days. After removing the medium, the six-well plates were washed twice with PBS. A total of 1mL of 4% paraformaldehyde was applied to each well for 30 min. After removing the 4% paraformaldehyde, the wells were dyed with a 0.1% crystal violet solution for 20 min. After cleaning with purified water, the cells on the six-well plates were photographed. The cells were counted using Image J software, and the pixel parameter was set to 20. All the experiments were repeated three times.

### 4.12. Migration Assay

Trypsin-digested cells were collected in 15 mL centrifuge tubes and centrifuged, and 30,000 cells were resuspended with 200 μL of serum-free medium and added to 8 µm-pore polycarbonate membrane transwell chambers (Corning, Corning, NY, USA) spiked with matrix gel (BD, Franklin Lakes, NJ, USA). The transwell lower chamber was supplemented with 500 μL of DMEM medium containing 20% serum and incubated in the incubator for 2 days before wiping off the non-migrating cells. This was followed by fixation with 4% paraformaldehyde for 10 min and staining with Giemsa for 30 min. Then, the cells were photographed using an inverted microscope (Olympus, Hatagaya, Japan). The cells were counted in 10 high-power microscope fields. Three images of each well were selected for each experiment and averaged. The cell counting was conducted using Image J software. All the experiments were repeated three times.

### 4.13. Spheroid Experiment

Without EDTA or phenol red, trypsin (Gbico, USA) was used to break down the cells. PBS applied three times and serum-free DMEM/F12 medium applied once were used to clean the cells. In total, 1000 cells were grown in serum-free DMEM/F12 with 20 ng/mL EGF and 10 ng/mL FGF on low-adhesion six-well plates (Corning, USA). The medium was added every three days and incubated continuously. The MCF-7 cell line was selected for 7 days of culture, while the Hs-578T cell line was selected to obtain a typical picture after 14 days of culture. Then, the cells were photographed using an inverted microscope (Olympus, Japan). The cells were counted in 10 high-power microscope fields. The number of tumor spheres larger than 50 μm in diameter was recorded using an inverted microscope and statistically analyzed. All the experiments were repeated three times.

### 4.14. Nude Mouse Xenograft Model

Female BALB/c nude mice aged four weeks were randomly separated into two groups. Sh-NC-MCF-7 and sh-NC-Hs-578T cells were implanted into the mice in the normal control group. The NUF2-knockdown mice were injected with sh-NUF2-Huh-7 and sh-NUF2-Hs-578 T cells. Each mouse received a 0.2 mL cell solution containing 1107 cells injected into the left abdomen. The tumor diameters were assessed on a weekly basis using digital calipers. After 28 days, the mice were euthanized. The tumor tissue was then excised, imaged, and weighed.

### 4.15. Statistical Analyses

The statistical work was performed in R (version 4.1.0) (George Ross Ihaka and Robert Gentleman, Oakland, New Zealand) and SPSS (version 23.0) (Norman H. Nie, C. Hadlai (Tex) Hull and Dale H. Bent, CA, USA). The MCC method was used to examine the important subnetworks using Cytoscape. Student’s *t*-test was used to analyze the TCGA database, together with fluorescence quantitative PCR and Western blot for the gene expression levels. For the study, Pearson’s correlation coefficient was employed. When the *p*-value of the differences was less than 0.05, we concluded that there was a statistically significant difference.

## Data Availability

The datasets used or analyzed during the current study are available from the corresponding author on reasonable request.

## Supplementary Materials

The following supporting information can be downloaded at: https://www.mdpi.com/article/10.3390/ijms24044226/s1.
