# Supplementary material for: NUF2 Promotes Breast Cancer Development as a New Tumor Stem Cell Indicator
Source: Int J Mol Sci. 2023 Feb 20;24(4):4226. doi: 10.3390/ijms24044226 (PMC9965662; doi:10.3390/ijms24044226)
Supplement: Supplementary file 1 [file ijms-24-04226-s001.zip › ijms-2050528-supplementary.pdf]

Table S1

Name	Abbreviation
Bladder Urothelial Carcinoma	BLCA
Breast invasive carcinoma	BRCA
Cholangiocarcinoma	CHOL
Colon adenocarcinoma	COAD
Esophageal carcinoma	ESCA
Head and Neck squamous cell carcinoma	HNSC
Kidney renal clear cell carcinoma	KIRC
Kidney renal papillary cell carcinoma	KIRP
Liver hepatocellular carcinoma	LIHC
Lung adenocarcinoma	LUAD
Lung squamous cell carcinoma	LUSC
Prostate adenocarcinoma	PRAD
Rectum adenocarcinoma	READ
Stomach adenocarcinoma	STAD
Thyroid carcinoma	THCA
Uterine Corpus Endometrial Carcinoma	UCEC

Table S2 The primers Sequences

Gene name	Sequences 5`→3`
KLF4(F)	CAGCTTCACCTATCCGATCCG
KLF4(R)	GACTCCCTGCCATAGAGGAGG
c-MYC(F)	GCCAACTACTCTACTGTGGATT
c-MYC(R)	ATGCAGATGACTGTGTCGTT
OCT4(F)	GCTCGAGAAGGATGTGGTCC
OCT4(R)	CGTTGTGCATAGTCGCTGCT
NANOG(F)	ACCTATGCCTGTGATTTGTGG
NANOG(R)	AGTGGGTTGTTTGCCTTTGG
NUF2(F)	GGAAGGCTTCTTACCATTCAGC
NUF2(R)	GACTTGTCCGTTTTGCTTTTGG
GAPDH(F)	ACATCGCTCAGACACCATG
GAPDH(R)	TGTAGTTGAGGTCAATGAAGGG

Table S3 sh-NUF2 Sequences

Name	Sequence
sh-NUF2-1	CGAATGGAAGTATCAGAAGTA
sh-NUF2-2	GAAGTATCAGAAGTACCAAAT
sh-NUF2-3	GAAGTCATGTATCCACATTTA
sh-NUF2-4	CTACATGAGAGCCTTACAAAT
